# Comparison of Measures of Ability in Adolescents with Intellectual Disability

**DOI:** 10.3389/fpsyg.2016.00683

**Published:** 2016-05-17

**Authors:** Chantanee Mungkhetklang, Sheila G. Crewther, Edith L. Bavin, Nahal Goharpey, Carl Parsons

**Affiliations:** ^1^Department of Psychology and Counselling, La Trobe UniversityMelbourne, VIC, Australia; ^2^Port Phillip Specialist SchoolPort Melbourne, VIC, Australia

**Keywords:** intellectual disability, autism spectrum disorder, adolescents, Wechsler Intelligence Scale for Children-Fourth Edition, Raven's Colored Progressive Matrices, Test of Non-verbal Intelligence-Fourth Edition, Wechsler Non-verbal Scale of Ability

## Abstract

Finding the most appropriate intelligence test for adolescents with Intellectual Disability (ID) is challenging given their limited language, attention, perceptual, and motor skills and ability to stay on task. The study compared performance of 23 adolescents with ID on the Wechsler Intelligence Scale for Children-Fourth Edition (WISC-IV), one of the most widely used intelligence tests, and three non-verbal IQ tests, the Raven's Colored Progressive Matrices (RCPM), the Test of Non-verbal Intelligence-Fourth Edition and the Wechsler Non-verbal test of Ability. Results showed that the WISC-IV Full Scale IQ raw and scaled scores were highly correlated with total scores from the three non-verbal tests, although the correlations were higher for raw scores, suggesting they may lead to better understanding of within group differences and what individuals with ID can do at the time of assessment. All participants attempted more questions on the non-verbal tests than the verbal. A preliminary analysis showed that adolescents with ID without ASD (*n* = 15) achieved higher scores overall than those presenting with ID+ASD (*n* = 8). Our findings support the view that short non-verbal tests are more likely to give a similar IQ result as obtained from the WISC-IV. In terms of the time to administer and the stress for participants, they are more appropriate for assessing adolescents with ID.

## Introduction

Assessments of intellectual ability occur at all stages of life for all levels of ability and society as the basis of clinical and education decisions, and for research purposes (Davis et al., 2000; Bradley-Johnson, 2001; Bittles et al., 2002; Heffer et al., 2009; Larsen et al., 2011; Baron and Leonberger, 2012). Such assessments are critical for identification and diagnosis of individuals with neurodevelopmental disorders, especially those with Intellectual Disability (ID).

ID is defined by the Diagnostic and Statistical Manual of Mental Disorders: DSM-5 (American Psychiatric Association, 2013) as an IQ of 70 or below, lower than expected, adaptive functioning and presence from early childhood. The previous DSM classification (DSM-IV) (American Psychiatric Association, 1994) classified ID in degrees of severity based on adaptive functioning and IQ: Mild (50–55 to approximately 70; ~85% of the ID population), Moderate (35–40 to 50–55; ~10% of ID), Severe (20–25 to 35–40; 3–4% of ID), and Profound (below 20 or 25; 1–2% of ID). Approximately 50% of individuals diagnosed with ID also have co-morbid Autism Spectrum Disorder (ASD) (Matson et al., 1996; Matson and Shoemaker, 2009; Wilkins and Matson, 2009), and Baio (2012) reported an estimated 31% of children with ASD have co-occurring ID with an additional 23% having IQs in the borderline range (71–84) (Baio, 2012). This confirms earlier observations by Vig and Jedrysek (1999) who reported that the more severe the individual's ID, the greater likelihood of ASD symptoms. It has also been shown that impaired verbal and non-verbal communication in individuals with ID co-morbid with ASD (ID+ASD) is more common than in those with ASD alone (Deb and Prasad, 1994).

Assessment scores for individuals with ID play a significant role in the allocation of clinical and educational services and monitoring of efficacy of training programs. However, intelligence testing in individuals with ID has significant limitations. Particular tests have emerged as the gold standard (e.g., the Wechsler Scales) often with insufficient up-to-date consideration or justification of the appropriateness of individual items and the scoring scheme for this population (Wechsler, 1949, 1974, 1997, 2003). Traditionally, measures of progress of cognitive development in ID have been made in terms of language-based items by comparing ID to Typically Developing (TD) groups of the same chronological and mental ages (Carroll, 1997). However, individuals with TD have vastly better verbal skills, raising the question of the most optimal and sensitive testing criteria.

Classical assessment tools rarely provide sensitive measurement for the low functioning individuals. For example, most intelligence tests do not measure IQ below 40 (e.g., the Wechsler Scales, Wechsler, 1949, 1974, 1997, 2003), the Kaufman Assessment Battery for children (Roid and Barram, 2004) and the Stanford Binet Scales (Thorndike et al., 1986), and so their use in measuring intellectual functioning below the mild ID range is limited. In addition, the normative samples rarely include an adequate number of participants with ID needed to provide sensitive measurement in the very low ability range, although they have been recruited for separate validation studies (Wechsler, 1949, 1974, 1997, 2003). Thus, floor effects have been discussed, particularly for different versions of the Wechsler Scales, e.g., Whitaker and Wood (2008) who made the recommendation to extrapolate the relationship between subtest raw scores and scaled scores below a scaled score of one.

Other researchers have addressed the issue by using re-standardized raw scores based on their sample specific statistic, but this confounds comparison of results across study samples. Hessl et al. (2009), Sansone et al. (2014), Benson et al. (2015) and Orsini et al. (2015) have suggested new scoring approaches to use for ID populations, e.g., Z-scores or the deviation Z-scores from raw scores for the Wechsler Intelligence Scale for Children-Fourth Edition (WISC-IV). However, the WISC-IV scaled score continues to be used to assess the cognitive abilities of different age groups and abilities, e.g., ranging from a 6-year-old child with moderate ID to a 16-year-old with intellectual giftedness or a TD individual, to plan special school programs (Sattler, 2008). Sattler (2008) has also suggested that individuals who are extremely high functioning are not adequately assessed on the WISC-IV and nor are those who are extremely low functioning. Individuals with ID usually have poor verbal ability, although usually more than expected from measures of their non-linguistic cognitive ability (Rondal, 2001; van der Schuit et al., 2011). Thus, reliance on scores from the verbal component may not be valid or a reliable measure of assessment of cognitive ability and may lead to a misunderstanding of their cognitive potential (Courchesne et al., 2015).

Because of the limitations associated with using the WISC-IV, alternative assessments using non-verbal measures have emerged as useful. Such non-verbal measures include the Raven's Colored Progressive Matrices (RCPM) (Raven et al., 1995), the Test of Non-verbal Intelligence-fourth edition (TONI-4) (Brown et al., 2010), the Comprehensive Test of Non-verbal Intelligence-Second Edition (CTONI-2) (Hammill et al., 2009), and the Wechsler Non-verbal Scale of Ability; (WNV) (Wechsler and Naglieri, 2006). The RCPM is well-known and has been much used cross-culturally (McCallum and SpringerLink, 2003) since its appearance in 1938 (Raven, 1938). Similarly to the RCPM, a low degree of cultural loading and linguistic demand is usually associated with the TONI-4 and the WNV (McCallum, 2013). In the current study we examined these three non-verbal tests. They were chosen on the basis of visual problem solving, less verbal expression required in administration and no need for verbal expression for responses.

This study firstly aimed to compare the raw scores (controlling for age) and scaled scores of an adolescent group with ID on the WISC-IV. Secondly we compared both scoring systems of the WISC- IV with the raw scores obtained on the three non-verbal tests as a means of establishing whether the non-verbal tests are adequate measures of cognitive ability in individuals with ID. That is, we investigated:

1. i. whether the WISC-IV scaled scores of our ID sample were similar to those of the ID sample reported in the manual;
2. ii. whether the RCPM, TONI-4, and WNV raw scores correlated significantly with the WISC-IV raw scores; and
3. iii. whether the WISC-IV raw scores or scaled scores are more appropriate for measuring cognitive understanding in individual with ID.

In addition, since it is well accepted that many adolescents with ID also show symptoms of ASD, we did a preliminary comparison of the raw scores on each test of those participants with ID and no diagnosis of ASD and those with co-morbid ID and ASD.

## Methods

### Participants

Participants were 23 adolescents (15 male and 8 female) with ID who attended a Specialist School in Melbourne, Australia. All had been clinically diagnosed prior to starting school with ID, and often a more specific neurodevelopmental disorder, by a trained clinician using medical records and the DSM-IV criteria. All had IQ scores below 70 at school enrollment. The mean age was 14 years and 1 month (range from 11 years and 3 months to 17 years 0 months; *SD* = 1.86) at the start of the study. Of the entire ID sample of 23, the largest subgroup was 8 (34.8%) who had previously been assessed by a panel of three clinical experts, as required by the State Government of Victoria, to meet the criteria for a clinical diagnosis of ASD. This group is referred to as the ID+ASD group. Another 15 had a primary diagnosis of ID, and included 3 with Attention Deficit and Hyperactive Disorder (13.0%), 1 with Down Syndrome (4.3%), 1 with Williams Syndrome (4.3%), 5 with Idiopathic ID (21.7%) and 5 with other medical diagnoses leading to ID (21.7%). One participant completed only 10 subtests of the 15 subtests of the WISC-IV but completed all three non-verbal tests and was included in the analyses where appropriate.

All parents/guardians provided informed consent prior to their child's participation. All individuals were screened for normal hearing and vision. Ethics approval was obtained from La Trobe University Human Ethics Committee and the State Department of Education. Permission to conduct testing in schools was obtained from the school principal.

### Materials

#### The Wechsler intelligence scale for children fourth edition (WISC-IV) (Wechsler, 2003)

The WISC-IV provides a Full-Scale IQ (FSIQ) to represent a child's overall cognitive ability (Flanagan and Kaufman, 2004). The WISC-IV can be used to assess individuals aged 6 years 0 months to 16 years 11 months. It has 10 core and 5 supplemental subtests. The subtests can be clustered into composite quotients for four indices. The subtests are grouped into four factors as follows: (a) the Verbal Comprehension Index (VCI) which is based on Similarity, Vocabulary, and Comprehension subtests; (b) the Perceptual Reasoning Index (PRI) which is based on the Block Design, Matrix Reasoning, and Picture Concepts subtests; (c) the Working Memory Index (WMI) which is based on the Digit Span and Letter-Number Sequencing subtests; and the Processing Speed Index (PSI) which is based on the Coding and Symbol Search subtests. The FSIQ is computed from all 10 core subtests. Internal consistency reliability coefficients range from 0.96 to 0.97 for the Full Scale IQ. Criterion validity with other intelligence tests range from 0.85 to 0.88 (Wechsler, 2003; Sattler, 2008).

See Table 1 for the WISC-IV subtests, index structure and task demands.

**Table 1:** **WISC-IV subtests, index structure and task demands**.

	**Verbal comprehension index**	**Perceptual reasoning index**	**Working memory index**	**Processing speed index**
Core subtest	Similarities	Block design	Digit span	Coding
	Vocabulary	Picture concept	Letter-number sequencing	Symbol search
	Comprehension	Matrix reasoning		
Supplemental subtest	Information	Picture completion	Arithmetic	Cancellation
	Word reasoning			
Task demands	Ability to listen to question, draw upon learned information from both formal and informal education, reason through an answer, and express thoughts.	Ability to examine a problem, draw upon visual-motor and visual-spatial skills, organize thoughts, create solutions, and then test them.	Ability to memorize new information, hold it in short-term memory, concentrate, and manipulate that information to produce some result or reasoning process.	Ability to focus attention and quickly scan, discriminate between and sequentially order visual information.

#### Raven's colored progressive matrices (RCPM) (Raven et al., 1995)

The RCPM is commonly used to obtain a non-verbal reasoning component and was designed for individuals aged 5 through 11 years, elderly persons, and mentally and physically impaired persons. The RCPM comprises 36 multiple-choice items of abstract reasoning divided into three subsets of 12 items. Each item consists of a different colored matrix pattern with a “missing” piece. Six possible pieces are displayed as alternatives to best complete the pattern. The puzzle version (Bello et al., 2008) was used in the current study as it has been shown to reliably measure non-verbal mentation of individuals equal to the standard book form in children with typical development (Bello et al., 2008), and to increase response rate of children with ID. The puzzle version of RCPM uses identical matrices to the book form, but the six alternative answers are attached by Velcro requiring the participant to actually replace and reattach the chosen/preferred answer. Only raw scores could be used for the RCPM because the manual does not report standard scores. Split-half reliabilities range from 0.65 to 0.94. Concurrent validity coefficients between Raven's Progressive Matrices and other intelligence tests are in the 0.50–0.80 range (Raven et al., 1995).

#### The test of non-verbal intelligence, 4th edition (TONI-4) (Banks and Franzen, 2010)

The TONI-4 is designed to assess problem-solving ability and abstract reasoning abilities of individuals aged 6–89 years. This test provides language-free measures of cognitive ability, and does not require reading or writing skill. Instructions are given via pantomime; participants respond by pointing, nodding or blinking. The TONI-4 Form A has five training items and 45 abstract/figural problem-solving items. Items are in a multiple-choice format, with either five or six response alternatives. Based on the TONI-3, it can differentiate individuals with ID from those without (Sattler, 2008). Internal consistency reliability scores are satisfactory, ranging from 0.94 to 0.97. Correlation coefficients with other non-verbal intelligence tests range from 0.73 to 0.79 (Brown et al., 2010).

#### The Wechsler non-verbal scale of ability (WNV) (Wechsler and Naglieri, 2006)

The WNV is an individually administered non-verbal test of intelligence designed for ages 4 through 21 years. The purpose of the WNV is to expand the clinical utility of the Wechsler scales to individuals with language constraints. The average internal consistency reliability for the Full Scale IQ on the four-subtests version is 0.91. Correlations between the Full Scale IQ using the four-subtest version and other tests of intelligence range between 0.71 and 0.82 (Wechsler and Naglieri, 2006).

### Procedure

The participants were tested individually on school grounds during school hours. All tests were conducted using standardized instructions, scoring and interpretation. The results for each participant were considered a valid assessment, not impeded by behavioral or emotional factors. The RCPM was usually administered in the initial session, the TONI-4 in the second, the WNV in the third and the WISC-IV in the last. The RCPM, TONI-4, and WNV were completed in one session (approximately 10–45 min) while the WISC-IV was completed in 2–4 sessions, with a range of 10–30 min per testing session, depending on the child's concentration. All standard test scores were obtained using the norms from the relevant test manuals. Although six participants were chronologically over 16 years 11 months, their mental age was under 9 years. Thus, we used standard score equivalents of ceiling chronological age provided in the WISC-IV manual. All 6 subtests from the WNV were included, as were the 10 core subtests from the WISC-IV plus the 5 supplemental subtests.

## Results

Both raw scores and scaled scores from each test were first checked for normality and the results were acceptable. Our first analysis investigated whether the WISC-IV scaled scores of our sample (*n* = 23) were similar to those of the ID norming sample, aged 6–16 years, reported in the manual. Descriptive statistics (mean and standard deviation) for the WISC-IV scaled scores and FSIQ in our sample and ID sample in the WISC-IV manual are reported in Table 2. In general, mean scores on all subtests, composites and FSIQ on the WISC-IV were slightly lower for our sample. However, there were no significant differences between our sample and the norming sample on any of the WISC-IV scaled score results. The subtests for which the two groups had the lowest and highest scores were the same: Arithmetic was the lowest and Cancellation the highest.

**Table 2:** **Comparison of WISC-IV Scaled Scores for the norming sample and the current sample**.

	**ID sample in the current study (*****n*** = 23**)**		**ID sample in the manual (*****n*** = 40**)**		**Mean difference of scaled scores of two samples**
	***M* (Range)**	***SD***	***M***	***SD***	
BD	4.1 (1–10)	2.6	4.7	2.8	0.6
SI	3.3 (1–8)	2.6	4.5	2.5	1.2
DS	4.0 (1–11)	3.2	5.4	2.9	1.4
PCn	3.1 (1–10)	2.9	5.1	2.9	2.0
CD	3.5 (1–8)	2.7	4.7	3.1	1.2
VC	3.8 (1–8)	2.5	4.9	2.1	1.1
LN	3.2 (1–8)	2.7	4.1	2.7	0.9
MR	4.1 (1–10)	2.8	4.4	2.7	0.3
CO	3.3 (1–7)	2.4	4.8	2.9	1.5
SS	3.5 (1–11)	3.2	4.8	2.5	1.3
PCm	3.8 (1–9)	2.6	4.3	2.8	0.5
CA	5.1 (1–13)	3.9	6.5	3.0	1.4
IN	3.4 (1–9)	2.7	4.4	2.1	1.0
AR	2.1 (1–6)	1.4	3.5	2.1	1.4
WR	3.5 (1–7)	2.1	5.2	2.4	1.7
VCI	62.0 (45–85)	14.5	69.3	13.2	7.3
PRI	61.9 (45–86)	13.8	67.7	14.4	5.8
WMI	63.9 (50–97)	14.5	70.2	13.3	6.3
PSI	64.6 (50–97)	14.9	71.2	13.4	6.6
FSIQ	55.6 (40–79)	13.1	62.9	12.1	7.3

BD, Block Design; SI, Similarities; PCn, Picture Concept; CD, Coding; VC, Vocabulary; LN, Letter-Number sequencing; MR, Matrices Reasoning; CO, Comprehension; SS, Symbol Search; PCm, Picture Completion; CA, Cancellation; IN, Information; AR, Arithmetic; WR, Word Reasoning; VCI, Verbal Comprehension Index; PRI, Perceptual Reasoning Index; WMI, Working Memory Index; PSI, Processing Speed Index; FSIQ, Full Scale IQ.

Descriptive statistics (mean and standard deviation) for the WISC-IV raw scores and scaled scores in our sample are reported in Table 3. There was a floor effect in all subtests of the WISC-IV. For example, the Block Design raw scores that ranged from 0 to 10 were converted to scaled scores as 1 (*n* = 9), the Vocabulary raw scores that ranged from 0 to 9 were converted to scaled scores as 1 (*n* = 6), the Arithmetic raw scores that ranged from 1 to 9 were converted to scales scored as 1 (*n* = 11) etc. Of the sample, only 7 participants did not have at least one scaled score of 1. Picture Concept, Letter-Number sequencing and Arithmetic showed the highest number of floor effects (*n* = 11). Letter-Number sequencing and Arithmetic are two of the three subtests of the Working Memory Index.

**Table 3:** **Raw and scaled WISC-IV scores for study sample**.

	**Raw scores**		**Scaled scores**	
	***M* (Range)**	***SD***	***M* (Range)**	***SD***
BD	20.1 (2–50)	13.4	4.1 (1–10)	2.6
SI	9.5 (0–27)	9.6	3.3 (1–8)	2.6
DS	10.1 (2–20)	4.6	4.0 (1–11)	3.2
PCn	10.1 (1–20)	5.3	3.1 (1–10)	2.9
CD	29.9 (4–57)	15.5	3.5 (1–8)	2.7
VC	20.6 (0–39)	11.1	3.8 (1–8)	2.5
LN	10.3 (0–18)	5.1	3.2 (1–8)	2.7
MR	13.6 (4–24)	6.2	4.1 (1–10)	2.8
CO	12.5 (0–24)	8.4	3.3 (1–7)	2.4
SS	14.5 (0–32)	9.3	3.5 (1–11)	3.2
PCm	17.7 (8–29)	6.6	3.8 (1–9)	2.6
CA	62.7 (12–115)	29.4	5.1 (1–13)	3.9
IN	11.9 (4–18)	4.1	3.4 (1–9)	2.7
AR	10.1 (1–20)	5.5	2.1 (1–6)	1.4
WR	9.2 (1–55)	3.8	3.5 (1–7)	2.1
VCI	42.6 (2–81)	26.7	62.0 (45–85)	14.5
PRI	43.9 (10–81)	22.0	61.9 (45–86)	13.8
WMI	20.4 (2–38)	8.9	63.9 (50–97)	14.5
PSI	44.4 (8–89)	22.6	64.6 (50–97)	14.9
TS	151.3 (30–264)	67.6	55.6 (40–79)	13.1

BD, Block Design; SI, Similarities; DS, Digit Span; PCn, Picture Concept; CD-B, Coding B; VC, Vocabulary; LN, Letter–Number sequencing; MR, Matrices Reasoning; CO, Comprehension; SS, Symbol Search; PCm, Picture Completion; CA, Cancellation; IN, Information; AR, Arithmetic; WR, Word Reasoning; VCI, Verbal Comprehension Index; PRI, Perceptual Reasoning Index; WMI, Working Memory Index; PSI, Processing Speed Index; TS, Total Score.

Pearson's correlation was used to compare the WISC-IV raw scores (co-varied for age) and scaled scores from the 10 core and 5 supplemental subtests. Associations among the subtests, composite and FSIQ of the WISC-IV are shown in Table 4. Both the raw scores controlling for age and the scaled scores of each subtest of the WISC-IV were highly correlated with the FSIQ, although inter-correlations among these subtests varied. Using raw scores, the Block Design correlated significantly with 5 of the 15 subtests, and using scaled scores with 4 of the 15 subtests. In the majority of cases the raw scores controlling for age showed higher correlations among the different tasks than when using scaled scores. For example, for Block Design raw scores controlling for age the correlation with FSIQ was higher (*r* = 0.657, *p* = 0.001) than when using scaled scores (*r* = 0.588, *p =* 0.003). Block Design controlling for age significantly correlated with the Perceptual Reasoning Index: raw scores *r* = 0.947, *p* < 0.001 and scaled scores, *r* = 0.801, *p* < 0.001. Block Design controlling for age significantly correlated with Matrix Reasoning: raw scores *r* = 0.814, *p* < 0.001 and scaled scores, *r* = 0.784, *p* < 0.001. For some subtests that showed significant correlations when using raw scores there were no significant correlation when scaled scores were used, and vice versa.

**Table 4:** **Correlations of Raw and Scaled WISC–IV component scores**.

	**BD**		**IS**		**DS**		**PCn**		**CD–B**		**VC**		**LN**		**MR**		**CO**		**SS**		**PCm**		**CA**		**IN**		**AR**		**WR**	
	**RS**	**SS**	**RS**	**SS**	**RS**	**SS**	**RS**	**SS**	**RS**	**SS**	**RS**	**SS**	**RS**	**SS**	**RS**	**SS**	**RS**	**SS**	**RS**	**SS**	**RS**	**SS**	**RS**	**SS**	**RS**	**SS**	**RS**	**SS**	**RS**	**SS**
SI																														
DS			^**^	^*^																										
PCn	^*^		^**^	^*^	^*^																									
CD	^**^	^**^	^*^		^**^		^**^																							
VC			^**^	^**^	^**^	^**^	^**^	^**^	^*^	^*^																				
LN			^**^	^**^	^**^	^**^	^*^		^**^	^*^	^**^	^**^																		
MR	^**^	^**^					^**^	^*^	^**^	^**^		^*^																		
CO			^**^	^**^	^**^	^**^	^**^	^*^	^*^	^*^	^**^	^**^	^**^	^**^																
SS		^*^					^**^	^**^	^**^	^**^		^*^	^*^		^**^	^**^	^**^	^**^												
PCm	^**^	^*^	^**^	^**^	^**^		^*^		^**^	^**^	^*^	^**^	^**^	^*^	^**^	^**^	^*^	^**^	^**^	^*^										
CA	^**^						^**^	^**^	^**^	^**^	^*^	^*^	^*^		^**^	^*^	^*^		^**^	^**^	^**^	^*^								
IN			^**^	^**^	^**^	^*^	^*^		^**^	^**^	^**^	^**^	^**^	^**^		^*^	^**^	^*^		^*^	^**^	^**^	^*^							
AR			^**^		^**^	^*^	^**^	^*^	^**^	^*^	^**^	^**^	^**^	^**^			^**^	^*^	^*^	^*^	^**^		^**^	^*^	^**^	^*^				
WR			^**^	^**^	^**^	^*^	^**^	^**^	^*^	^*^	^**^	^**^	^**^	^**^	^*^	^*^	^**^	^**^	^*^	^**^	^**^	^**^	^**^	^*^	^**^	^**^	^**^	^**^		
VCI			^**^	^**^	^**^	^**^	^**^	^**^	^*^	^*^	^**^	^**^	^**^	^**^		^*^	^**^	^**^	^*^	^*^	^**^	^**^	^*^		^**^	^**^	^**^	^**^	^**^	^**^
PRI	^**^	^**^		^*^	^*^		^**^	^**^	^**^	^**^		^**^			^**^	^**^			^**^	^**^	^**^	^**^	^**^	^**^		^*^	^*^			^**^
WMI			^**^	^**^	^**^	^**^	^*^		^**^	^*^	^**^	^**^	^**^	^**^			^**^	^**^			^**^	^*^	^*^		^**^	^**^	^**^	^**^	^**^	^**^
PSI	^**^	^**^	^*^		^*^		^**^	^*^	^**^	^**^	^*^	^*^	^**^	^*^	^**^	^**^	^**^	^**^	^**^	^**^	^**^	^**^	^**^	^**^	^**^	^*^	^**^	^*^	^**^	^*^
TS	^**^	^**^	^**^	^**^	^**^	^**^	^**^	^**^	^**^	^**^	^**^	^**^	^**^	^**^	^**^	^**^	^**^	^**^	^**^	^**^	^**^	^**^	^**^	^**^	^**^	^**^	^**^	^**^	^**^	^**^

BD, Block Design; SI, Similarities; DS, Digit Span; PCn, Picture Concept; CD, Coding; VC, Vocabulary; LN, Letter-Number sequencing; MR, Matrices Reasoning; CO, Comprehension; SS, Symbol Search; PCm, Picture Completion; CA, Cancellation; IN, Information; AR, Arithmetic; WR, Word Reasoning; VCI, Verbal Comprehension Index; PRI, Perceptual Reasoning Index; WMI, Working Memory Index; PSI, Processing Speed Index; TS, Total Score.

* p < 0.05,

** p < 0.01.

Our third investigation was to determine whether the RCPM, TONI-4, and WNV raw scores (controlling for age) correlated significantly with the WISC-IV raw scores. Descriptive statistics (mean and standard deviation) for the raw scores and scaled scores on the three non-verbal tests are reported in Table 5. Pearson correlation was again used to assess associations among the subtests and composites of the WISC-IV and three non-verbal tests. Table 6 shows the results.

**Table 5:** **Scores (mean and standard deviation) for three non-verbal tests in study sample**.

	**Raw scores**		T-/Standard scores^*^	
	***M* (Range)**	***SD***	***M* (Range)**	***SD***
RCPM	25.6 (13–34)	7.4	–	–
TONI–4	22.2 (9–36)	6.9	83.0 (68–107)	9.0
WNV				
MA	16.8 (5–25)	5.1	38.2 (10–53)	11.1
CD–A	36.83 (2–68)	19.18	–	–
CD–B	25.4 (1–51)	12.7	24.1 (10–42)	11.0
SS	10.6 (3–19	4.8	37.5 (18–61)	12.8
PA	7.8 (0–19)	7.0	34.1 (10–59)	14.6
OA	29.76 (3–50)	13.85	–	–
RC	14.78 (8–21)	3.79	–	–
TS	60.6 (9–105)	27.3	67.8 (33–103)	20.0

RCPM, Raven's Colored Progressive Matrices; TONI–4, Test of Non-verbal Intelligence–4th edition; WNV, Wechsler Non-verbal scale of ability; MA, Matrices, CD, Coding; SS, Spatial Span; PA, Picture Arrangement; OA, Object Assembly; RC, Recognition; TS, Total Score of the WNV. RCPM does not provide scaled scores. Three subtests of the WNV (Coding A, Object assembly and Recognition) were normed for young children aged 4–7 years. Our sample was aged 11–18 years.

* T–scores for the TONI–4 and Standard scores for the WNV.

**Table 6:** **Correlations between raw scores on the WISC–IV components and the three non-verbal tests**.

	**BD**	**SI**	**DS**	**PCn**	**CD–B**	**VC**	**LN**	**MR**	**CO**	**SS**	**PCm**	**CA**	**IN**	**AR**	**WR**	**VCI**	**PRI**	**WMI**	**PSI**	**TS96I**
RCPM	^**^	^**^	^**^	^**^	^**^	^*^		^**^	^*^	^**^	^**^	^**^	^**^	^**^	^**^	^**^	^**^	^**^	^**^	^**^
TONI–4	^**^	^*^		^**^	^**^			^**^		^*^	^*^	^*^	^*^				^**^		^**^	^**^
WNV_MA	^**^			^**^	^**^		^*^	^**^		^**^	^**^	^**^	^*^				^**^	^*^	^**^	^**^
WNV_CD–A	^*^		^*^	^*^	^*^	^*^		^**^	^*^	^**^	^**^	^**^	^*^	^*^	^**^	^**^	^**^	^*^	^**^	^*^
WNV_CD–B	^**^		^**^	^**^	^**^		^**^	^**^	^**^	^**^	^*^	^**^	^*^	^*^	^*^	^*^	^**^	^**^	^**^	^**^
WNV_SS	^**^	^*^	^*^	^**^	^**^	^**^	^**^	^**^	^*^	^**^	^**^	^**^	^*^	^**^	^**^	^**^	^**^	^**^	^**^	^**^
WNV_PA	^**^	^*^		^**^	^**^	^**^	^**^	^**^	^*^	^**^	^**^	^**^	^**^	^**^	^*^	^**^	^**^	^*^	^**^	^**^
WNV_OA	^**^	^*^	^*^	^**^	^**^	^*^	^**^	^**^	^*^	^**^	^**^	^**^	^**^	^**^	^**^	^**^	^**^	^**^	^**^	^**^
WNV_RC	^**^	^**^	^**^	^**^	^**^	^*^	^**^	^**^	^*^	^**^	^**^	^**^	^**^	^**^	^**^	^**^	^**^	^**^	^**^	^**^
WNV_TS–II	^**^	^*^	^*^	^**^	^**^	^**^	^*^	^**^	^**^	^*^	^**^	^**^	^**^	^**^	^*^	^**^	^**^	^**^	^**^	^**^

BD, Block Design; SI, Similarities; DS, Digit Span; PCn, Picture Concept; CD, Coding; VC, Vocabulary; LN, Letter–Number sequencing; MR, Matrices Reasoning; CO, Comprehension; SS, Symbol Search; PCm, Picture Completion; CA, Cancellation; IN, Information; AR, Arithmetic; WR, Word Reasoning; VCI, Verbal Comprehension Index; PRI, Perceptual Reasoning Index; WMI, Working Memory Index; PSI, Processing Speed Index; TS–I, Total Score of the WISC–IV; RCPM, Raven's Colored Progressive Matrices; TONI–4, Test of Non-verbal Intelligence–4th edition; WNV_MA, Matrices subtest of the WNV, WNV_CD–A, Coding A subtest of the WNV; WNV_CD–B, Coding B subtest of the WNV; WNV_SS, Spatial Span subtest of the WNV; WNV_PA, Picture Arrangement subtest of the WNV; WNV_OA, Object Assembly subtest of the WNV; WNV_RC, Recognition subtest of the WNV; WNV_TS-II, Total Score of the Wechsler Non-verbal scale of ability.

* p < 0.05,

** p < 0.01.

Significant correlations were found between the FSIQ of the WISC-IV and all the non-verbal tests, including each subtest of the WNV as well as non-verbal subtests of the WISC-IV. There were also significant correlations between the non-verbal tests and the verbal subtests of the WISC-IV: Similarities, Vocabulary, Comprehension, and Information. Raw scores controlling for age for the RCPM were significantly correlated with 14 out of the 15 subtests of the WISC-IV; Letter-Number sequencing was the only subtest that did not show a significant correlation. Raw scores for the TONI-4 significantly correlated with all subtests of the Perceptual Reasoning Index and the Processing Speed Index. The TONI-4 scores also showed significant correlations with Similarities and Information of the Verbal Comprehension Index, but there were no significant correlations with any of the subtests of the Working Memory Index. Raw scores for each subtest of the WNV significantly correlated with all subtests of the Perceptual Reasoning Index and Processing Speed Index. All subtests of the WNV except Matrices significantly correlated with the Verbal Comprehension Index and Working Memory Index. Only the Information subtest of the Verbal Comprehension Index of the WISC-IV showed significant correlations with all non-verbal tests, including all subtests in the WNV.

As reported in the introduction, ID is often co-morbid with ASD and certainly there were many and varied clinical diagnoses for the adolescents with ID in our sample. The largest subgroup (co-morbid ID+ASD) included only 8 individuals and so we compared the pattern of performance of those adolescents with that of the participants without ASD (*n* = 15) using non-parametric statistics. Means and standard deviations for the raw scores and the scaled scores for the WISC-IV, RCPM, TONI-4, and WNV for the two groups are reported in Table 6. On the WISC-IV, Matrix Reasoning had the highest mean scaled scores for the ID+ASD group, while Cancellation was the highest for the non-ASD group. As shown in Table 7, scores on Arithmetic were the lowest for both groups. The Similarities subtest was also low for the ID+ASD group. The ID+ASD scores were slightly higher on some of the non-verbal tasks (Block Design, Coding, Matrix Reasoning, and Picture Completion) than the non-ASD group.

**Table 7:** **Comparison of raw scores on all tests for ID Non–ASD and ID+ASD participants**.

	**ID+ASD (*****n*** = 8**)**		**ID Non–ASD (*****n*** = 15**)**		**Mean difference of two sample**			**Effect size**	
	***M* (Range)**	***SD***	***M* (Range)**	***SD***	**Difference**	***U***	***p***	**Cohen's *d***	***r***
**RCPM**	24.0 (13.32)	8.1	26.4 (13–34)	7.0	2.4	48.5	ns	0.31	0.15
**TONI–4**	21.6 (13–34)	7.7	22.5 (9–36)	6.8	0.9	54.5	ns	0.12	0.06
**WVN**									
WNV_MA	16.9 (5–25)	6.8	16.7 (11–23)	4.1	0.1	53.0	ns	0.03	0.02
WNV_CD A	35.75 (2–66)	24.8	37.4 (9–68)	16.4	1.6	59.0	ns	0.08	0.04
WNV_CD B	24.4 (1–42)	16.0	26.0 (4–51)	11.1	1.6	41.5	ns	0.04	0.02
WNV_SS	9.0 (3–15)	4.3	11.5 (4–19)	4.9	2.5	50.0	ns	0.54	0.26
WNV_PA	6.5 (0–14)	5.6	8.5 (0–9)	7.7	2.0	60.0	ns	0.29	0.14
WNV_OA	30.5 (9–46)	13.6	29.4 (3–50)	14.5	1.1	54.5	ns	0.08	0.04
WNV_RC	14.7 (8–18)	4.0	14.9 (8–21)	3.8	0.2	59.5	ns	0.05	0.02
WVN_TS-II	56.7 (9–90)	32.2	62.7 (21–105)	25.3	5.9	36.0	ns	0.21	0.10
**WISC–IV**									
BD	22.0 (2–50)	19.0	19.2 (2–31)	10.0	2.8	54.5	ns	0.18	0.09
SI	3.5 (0–27)	9.5	12.7 (0–23)	8.3	9.2	23.0	^*^	1.03	0.46
DS	7.7 (2–15)	4.1	11.5 (3–20)	4.5	3.7	27.5	^*^	0.88	0.40
PCn	9.0 (4–16)	4.0	10.7 (1–20)	6.0	1.7	48.5	ns	0.33	0.16
CD–B	29.1 (4–52)	17.4	30.3 (6–57)	15.0	1.2	54.0	ns	0.07	0.04
VC	10.5 (0–30)	9.2	25.9 (14–39)	7.9	15.4	11.5	^**^	1.79	0.67
LN	6.9 (0–16)	5.4	12.1 (6–18)	4.0	5.2	26.5	^*^	1.09	0.48
MR	14.2 (4–22)	7.4	13.2 (6–24)	5.6	1.0	53.0	ns	0.15	0.07
CO	6.9 (0–24)	9.0	15.5 (1–24)	6.6	8.6	27.0	^*^	1.09	0.48
SS	13.7 (0–28)	9.6	14.9 (1–32)	9.8	1.2	56.5	ns	0.12	0.07
PCm	16.5 (8–29)	7.8	17.2 (0–27)	7.5	1.9	52.5	ns	0.09	0.04
CA	53.4 (12–90)	29.0	63.5 (0–115)	33.2	14.6	49.0	ns	0.32	0.16
IN	8.9 (4–16)	3.8	12.7 (0–18)	4.6	4.7	25.5	^*^	0.09	0.41
AR	6.6 (4–17)	4.8	11.3 (0–20)	6.0	5.4	32.0	ns	0.86	0.40
WR	6.0 (1–13)	4.1	10.3 (0–15)	3.5	5.0	24.0	^*^	1.23	0.49
VCI	20.9 (2–81)	25.8	54.1 (21–80)	19.4	33.2	18.5	^**^	1.45	0.59
PRI	45.2 (11–81)	27.4	43.1 (10–68)	19.7	2.1	58.8	ns	0.09	0.04
WMI	14.6 (2–31)	9.4	23.5 (9–38)	7.1	8.9	26.0	^*^	1.07	0.47
PSI	42.9 (8–74)	22.5	45.3 (7–89)	23.5	2.4	56.0	ns	0.10	0.05
TS–I	123.6 (30–240)	71.0	166.1 (58–264)	63.2	42.4	36.0	ns	0.63	0.30

BD, Block Design; SI, Similarities; DS, Digit Span; PCn, Picture Concept; CD, Coding; VC, Vocabulary; LN, Letter–Number sequencing; MR, Matrices Reasoning; CO, Comprehension; SS, Symbol Search; PCm; Picture Completion; CA, Cancellation; IN, Information; AR; Arithmetic; WR, Word Reasoning; VCI, Verbal Comprehension Index; PRI, Perceptual Reasoning Index; WMI, Working Memory Index; PSI–Processing Speed Index; TS-I, Total Score of the WISC-IV; RCPM, Raven's Colored Progressive Matrices; TONI–4, Test of Non-verbal Intelligence–4th edition; WNV_MA, Matrices subtest of the WNV, WNV_CD–A, Coding A subtest of the WNV; WNV_CD–B, Coding B subtest of the WNV; WNV_SS, Spatial Span subtest of the WNV; WNV_PA, Picture Arrangement subtest of the WNV; WNV_OA, Object Assembly subtest of the WNV; WNV_RC, Recognition subtest of the WNV; WNV_TS-II, Total Score of the Wechsler Non-verbal scale of ability.

* p < 0.05,

** p < 0.01; ns, not significant.

Based on Mann-Whitney U comparisons, there was no significant difference between the ID non-ASD and ID+ASD for the RCPM, the TONI-4 and the WNV. However, there were significant group difference between the ID non-ASD and ID+ASD for some of the subtest scores on the WISC-IV: Similarities (*p* = 0.015), Digit Span (*U* = 23, *p* = 0.034), Vocabulary (*U* = 11.5, *p* = 0.002), Letter-Number Sequencing (*U* = 26.5, *p* = 0.030), Comprehension (*U* = 27, *p* = 0.033), Information (*U* = 25.5, *p* = 0.025) and Word Reasoning (*U* = 24, *p* = 0.020). Furthermore, there were significant group difference between the ID non-ASD and ID+ASD for the Verbal Comprehension Index (*U* = 18.5, *p* = 0.007) and Working Memory Index (*U* = 26, *p* = 0.028) on the WISC-IV composite score.

Pearson correlation was used to assess associations among the raw scores of the subtests, the composite and the total raw scores of the WISC-IV and three non-verbal tests for the ID non-ASD group (see Table 8) and the ID+ASD group (see Table 9).

**Table 8:** **Correlations between raw scores from each WISC–IV subtest and three non-verbal tests for the ID non–ASD (*n* = 15)**.

	**BD**	**SI**	**DS**	**PCn**	**CD–B**	**VC**	**LN**	**MR**	**CO**	**SS**	**PCm**	**CA**	**IN**	**AR**	**WR**	**VCI**	**PRI**	**WMI**	**PSI**	**TS–I**
RCPM	**	*	*		**	**	*	**		*	**	*	*	*	**	**	**	**	**	**
TONI–4	**			*				*		*	*		**		*		**		*	*
WNV_MA	**	**		*	**	**		**		**	**	**	**	*	**	**	**		**	**
WNV_CD–A	**	**		*	**	*	*	**	**	**	**	**	*	**	*	**	**	*	**	**
WNV_CD–B	**			*	**		*		**	**	*	**	*	**		*	**	*	**	**
WNV_SS	**			**	**	*		*		**	*	**		**	*	*	**		**	**
WNV_PA	**	*		*	**	**	*	**		**	**	**	**	*		**	**		**	**
WNV_OA	**	**		*	**	*	*	**		**	**	**	*		**	**	**		**	**
WNV_RC	**			*	*	**		**	*	*	*		**	*	**	**	**		**	**
WNV_TS–II	**	*		**	**	**	**	**	*	**	**	**	**	**	*	**	**	*	**	**

**Table 9:** **Correlations between raw scores from each WISC–IV subtest and three non-verbal tests for the ID+ASD (*n* = 8)**.

	**BD**	**SI**	**DS**	**PCn**	**CD–B**	**VC**	**LN**	**MR**	**CO**	**SS**	**PCm**	**CA**	**IN**	**AR**	**WR**	**VCI**	**PRI**	**WMI**	**PSI**	**TS–I**
RCPM	^**^				^**^			^**^				^*^					^**^		^**^	^**^
TONI–4	^*^				^*^			^*^									^**^			^*^
WNV_MA	^*^				^**^			^**^									^**^		^**^	^*^
WNV_CD–A	^*^		^*^		^**^			^**^			^*^						^**^	^*^	^**^	^**^
WNV_CD–B	^*^		^*^		^**^			^**^									^**^		^**^	^*^
WNV_SS	^*^		^*^		^**^			^**^			^*^	^*^					^**^	^*^	^**^	^**^
WNV_PA	^*^				^**^			^**^				^*^					^**^		^**^	^**^
WNV_OA	^*^				^*^			^**^				^*^					^**^		^*^	^**^
WNV_RC																				
WNV_TS–II	^*^		^*^		^**^			^**^			^*^	^*^					^**^	^*^	^**^	^**^

BD, Block Design; SI, Similarities; DS, Digit Span; PCn, Picture Concept; CD, Coding; VC, Vocabulary; LN, Letter–Number sequencing; MR, Matrices Reasoning; CO, Comprehension; SS, Symbol Search; PCm; Picture Completion; CA, Cancellation; IN, Information; AR; Arithmetic; WR, Word Reasoning; VCI, Verbal Comprehension Index; PRI, Perceptual Reasoning Index; WMI, Working Memory Index; PSI–Processing Speed Index; TS-I, Total Score of the WISC-IV; RCPM, Raven's Colored Progressive Matrices; TONI–4, Test of Non-verbal Intelligence–4th edition; WNV_MA, Matrices subtest of the WNV, WNV_CD–A, Coding A subtest of the WNV; WNV_CD–B, Coding B subtest of the WNV; WNV_SS, Spatial Span subtest of the WNV; WNV_PA, Picture Arrangement subtest of the WNV; WNV_OA, Object Assembly subtest of the WNV; WNV_RC, Recognition subtest of the WNV; WNV_TS-II, Total Score of the Wechsler Non-verbal scale of ability.

* p < 0.05,

** p < 0.01.

For the ID non-ASD group, all three non-verbal tests significantly correlated with the WISC-IV FSIQ. The RCPM was significantly correlated with all subtests of the Verbal Comprehension Index of the WISC-IV: Similarities, Vocabulary, Information and Word Reasoning, while the TONI-4 was significantly correlated with only one subtest, Information. For the ID+ASD group, none of the Verbal Comprehension Index subtests of the WISC-IV were significantly correlated with any of the non-verbal tests.

## Discussion

The purpose of this study was to examine performance of an adolescent group with ID on the WISC-IV and on three non-verbal tests: the RCPM, the TONI-4 and the WNV. The most important result in this study is that both the raw scores (controlling for age) and the scaled scores showed highly significant correlations across the different measures (the WISC-IV, RCPM, TONI-4, and WNV), in particular between the WISC-IV FSIQ and the three non-verbal tests. Not surprisingly, performance measured using the scaled scores indicated that all individuals with ID performed well below average for their chronological age on all four tests. Our results also showed no statistically significant differences between our sample and the ID norming sample on the scaled scores of each subtest, composite scores and FSIQ. Interestingly, however, for our sample both WISC-IV scaled scores and raw scores controlling for age were highly correlated with FSIQ scores. The correlations were higher for the raw scores, suggesting that the raw scores of the WISC-IV are the optimal for measuring reasoning ability for individuals with ID; this is because the scaled scores reflect floor effects.

Our finding of such high significant correlations across the different measures of IQ (WISC-IV, RCPM, TONI-4, and WNV), especially between the WISC-IV FSIQ and the three non-verbal tests, is very important both operationally, in terms of establishing better assessment procedures for individuals with ID, and theoretically, in terms of how generalized the concept of cognitive functioning is. Given that there are many conceptual and procedural differences between the four tests in term of design, structure, and language requirements both during administration and in terms of responses, and time needed for completion, it is of importance that we found comparable full scaled IQ results on all four tests. The adolescents with ID performed particularly poorly on tasks requiring verbal reasoning but performed relatively better on non-verbal tasks, suggesting, firstly, that one of the non-verbal short tests would be a more appropriate tool to measure potential cognitive performance in individuals with ID given their well-documented short attention span (Chakrabarti and Banerjee, 2013). Secondly our results suggest that, at least for our sample, cognitive performance is limited by verbal skills. Additionally, the maximum non-verbal mental age an individual with ID (IQ less than 70 on the WISC-IV) is likely to attain eventually in each subtest is a raw score representative of approximately 8 chronological years, or lower, of typical development (Wechsler, 2003).

There are pragmatic advantages in using a non-verbal IQ test. The WISC-IV usually requires 60–90 min (according to the WISC-IV manual) or much longer for intellectually disabled individuals (Wechsler, 2003). Our ID group completed the WISC-IV within 2 to 4 sessions. By contrast, each of the three non-verbal tests only took one session, with the RCPM taking approximately 13 min, the TONI-4 15 min and the WNV 45 min. Thus, we would argue that the language requirements and time to administer the WISC-IV make the non-verbal tests, especially the RCPM or TONI-4, better options than the WISC-IV or the WNV.

The inability of many of the participants to perform some subtests of the WISC-IV, including Vocabulary, and hence the associated floor effects also argues against the use of the WISC-IV and scaled scores, as suggested by Whitaker (2010) and Sansone et al. (2014). Intelligence tests with large floor effects typically have reduced range and variability, and create positively skewed data. In the current study, significant correlations were found between all three non-verbal tests and the WISC-IV. The highest correlations were with the Perceptual Reasoning Index (Block Design, Picture Concept, Matrix Reasoning, and Picture Completion) and the Processing Speed Index (Coding, Symbol Search, and Cancellation). These findings are consistent with the WISC-IV manual (Wechsler, 2003), which states that within-group comparisons for children with ID are expected to reveal slightly higher scores on the Processing Speed Index than on the Verbal Comprehension Index and Perceptual Reasoning Index.

Non-parametric analysis of scores on the subtests of the WISC-IV of the ID non-ASD group and those co-morbid for ID+ASD demonstrated that, although the verbal ability of all participants was poor, it was lower for those with ID+ASD. Adolescents with ID without co-morbid ASD were able to attempt some verbal subtests, e.g., Vocabulary, whereas all of the adolescents with ID+ASD were either limited to repeating the question or making no response. Thus, our findings with this small group support previous reports suggesting that the main impairment in individuals with both ID and ID+ASD is in the verbal domain (Vig and Jedrysek, 1999; Brereton et al., 2006; Wilkins and Matson, 2009; van der Schuit et al., 2011). Furthermore, the Matrix Reasoning subtest of the WISC-IV resembles some aspects of the Raven's Progressive Matrices (Wechsler, 2003) and so it was expected that our study would find that the highest subtest score for children with ID+ASD would be on the Matrix Reasoning subtest. A number of previous studies have noted that the Matrix Reasoning subtest of the WISC-IV is indicative of relative cognitive strength for individuals with ASD (Mayes and Calhoun, 2006, 2008). Other studies (Dawson et al., 2007) have suggested that while the WISC-III and the Raven's Standard Progressive Matrices (RSPM) provided similar estimates for children with TD, children with ASD perform significantly better on the RSPM. This finding is consistent with Barbeau et al. (2013) and Nader et al. (2014), who suggested that the RSPM non-verbal test may be a better choice than the Wechsler scales to assess children with ASD, and in comparing the RSPM and WISC-IV scores, Nader et al. (2014) showed that the WISC-IV FSIQ underestimated the IQ of individuals with ASD in that they achieved a significantly higher mental age on the RSPM.

## Conclusion

The results of this study have important implication for the assessment of individuals with ID with and without ASD, emphasizing the need for theoretical considerations of what IQ tests really measure. Our results indicate that the three short non-verbal tests (RCPM, TONI-4, WNV) compared here provide an adequate estimate of IQ. The value in using the lengthy verbal language-based WISC-IV is that it can provide separate verbal and non-verbal IQ scores if required, but a main disadvantage is the time taken to administer.

We also found that the use of raw scores controlling for age provided a more appropriate measure of WISC-IV performance in our limited ID sample, than did scaled scores. Furthermore, the use of raw scores is more likely to lead to better understanding of within group differences and what individuals with ID are capable of, at the time of assessment. Use of raw scores will facilitate further investigation of the type of errors individuals commonly make, and assessment of the problem solving strategies they habitually use. Such information will also be useful for planning individual programs aimed at accommodating the variability found in a group of individuals with ID in one class, and hopefully better encourage each individual to achieve their potential.

## Author contributions

CM—collected the data, conducted the analysis and interpretation of the data, and prepared the early drafts of the paper. SC—Supervised development of the research, data interpretation and manuscript preparation. EB—Supervised development of the research, data interpretation and preparation of the manuscript. NG—Collected the data and helped in preparation of the manuscript. CP—Helped in recruitment and provided testing facilities.

## Funding

The first author was supported by a Royal Thai Government PhD Scholarship and additional funds were provided by the School of Psychology and Public Health, La Trobe University.

### Conflict of interest statement

The authors declare that the research was conducted in the absence of any commercial or financial relationships that could be construed as a potential conflict of interest.
